# Exsolution of Ni nanoparticles in A-site excess STO films†

**DOI:** 10.1039/d4na00213j

**Published:** 2024-10-14

**Authors:** Kevin G. Both, Dragos Neagu, Øystein Prytz, Truls Norby, Athanasios Chatzitakis

**Affiliations:** a Centre for Materials Science and Nanotechnology, Department of Physics, University of Oslo Gaustadalléen 21 NO-0349 Oslo Norway; b Department of Chemical and Process Engineering, University of Strathclyde 75 Montrose St G1 1XJ Glasgow UK; c Centre for Materials Science and Nanotechnology, Department of Chemistry, University of Oslo Gaustadalléen 21 NO-0349 Oslo Norway k.g.both@smn.uio.no athanasios.chatzitakis@smn.uio.no

## Abstract

Exsolution is a technique to create metal nanoparticles embedded within a matrix. The phenomenon has previously predominantly been studied in A-site deficient and stoichiometric perovskite powders. Here, we present a systematic study of an A-site excess perovskite oxide based on SrTiO_3_ thin films, doped with nickel and exsolved under different conditions. The study aims to shed light on particle formation in these novel systems, including the effects of (i) the thin film thickness, (ii) pre-exsolution annealing in an oxidative atmosphere, (iii) a reductive atmosphere during the exsolution step, and (iv) exsolution time on the particle size and particle density. Our results indicate that exsolution occurs quickly, forming nanoparticles both on the surface and in the bulk of the host perovskite. The findings indicate that pre-annealing in an ambient atmosphere leads to fewer but larger exsolved particles compared to samples without pre-annealing. Consequently, while crystallization of the thin film occurs in both atmospheres, the simultaneous crystallization of the thin film and formation of the nanoparticles leads to a smaller apparent average radius. Moreover, we present evidence that metal particles can be found beyond the originally doped region. These findings are a step towards realizing tunable functional materials using exsolution to create metallic nanostructures within a thin film in a predictable manner.

## Abstract 2

We suggest ways to control the size of exsolved particles and to create regions of high particle density within materials.

## Graphical abstract

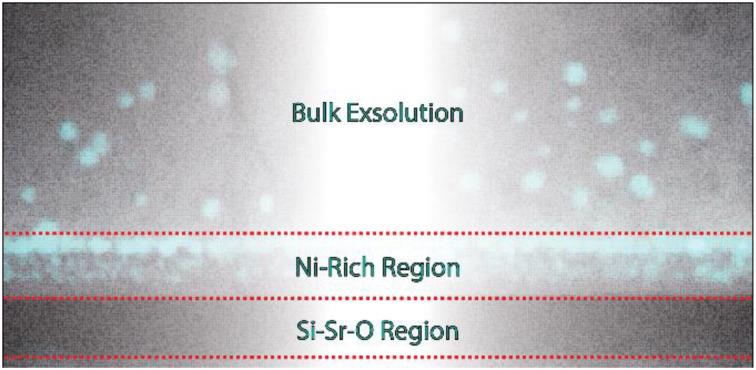

## 1. Introduction

The focus on metal nanoparticles (MNPs) in different structures has increased sharply in recent years, with applications across catalysis and photocatalysis, electrochemical energy devices, electronic, and optics.^1–8^ Exsolution has been utilized to create MNPs on oxide supports, and has been well-studied for A-site deficient and stoichiometric perovskite powders.^9–11^ The MNPs on the surface of such oxides are socketed, making them more stable and less prone to coking than their counterparts synthesized through techniques like chemical vapor deposition, electro(less) deposition, and impregnation.^1,12–14^ In brief, under oxidizing conditions, the B-site is partially substituted with transition metals. To compensate for the loss of oxygen under reducing conditions, the transition metals are exsolved, forming MNPs.

The mechanism of exsolution involves four steps: (i) cation and oxide ion diffusion, (ii) cation reduction, (iii) nucleation, and (iv) particle growth.^15^ The reduction of the perovskite lattice through removal of oxide ions leads to oxygen vacancies, destabilizing the perovskite structure and necessitating a decrease in the oxidation state of a subset of the cations, *i.e.*, towards a metallic state.^16^ The surface morphology during exsolution has been studied by Neagu *et al.*,^17^ who observed the process *in situ* and in real time showing that the socket and particle form together. The particle almost ruptures the surface and pushes it aside to facilitate its emergence. This leads to strain and socket formation. Moreover, the crystallographic facet of the perovskite influences the degree of exsolution, and generally, MNPs grow epitaxially on the perovskite substrate.^17^ MNP exsolution is not limited to the surface but also occurs in the bulk.^18,19^ Their presence in the bulk can drastically influence the properties of the materials, as shown by Kousi *et al.* in ref. 19 who demonstrated that the presence of MNPs in the bulk enhances the oxygen exchange across the nanocomposite. Wang *et al.* showed that the MNPs and exsolution-induced defects can alter the material's properties profoundly, *i.e.*, they observed a drastic improvement in electronic conductivity.^20^ Recently, Syed and co-workers published a study on various types of nanostructures synthesized *via* bulk exsolution in La_0.6_FeO_3_ thin films.^21^ They highlighted the need to systematically investigate the impact of synthesis and processing conditions on the MNP formation. Weber *et al.* showed spontaneous phase separation on the nanoscale which can result in the formation of Ni-rich nanostructures within the as-synthesized perovskite lattice. These domains may act as nucleation sites for MNPs.^22^

A-site excess perovskites frequently exhibit Ruddlesden–Popper (RP, *e.g.*, Sr_1+*n*_Ti_*n*_O_3*n*+1_) interlayers, accommodating the excess A site ions. Contrary to A-site deficient perovskites, A-site excess perovskites exhibit no inherent A-site vacancies, and only a limited presence of oxygen vacancies. This creates a material where exsolution is less thermodynamically driven by the change towards a stoichiometric perovskite than their A-site deficient counterparts.^23^ Conversely, kinetic improvements due to improved ion diffusion in the interlayer region may be observed in A-site excess perovskites, resulting in improved control over particle distribution and “socketing” depth on the surface of the host semiconductor.

We have recently investigated gold (Au) MNPs introduced by galvanically replacing exsolved nickel (Ni) particles on Ni-doped A-site excess strontium titanate (Sr_1+*x*_Ti_1−*x*_Ni_*x*_O_3±*δ*_, with *x* = 0.07, STNO).^18^ There we detected a difference in the metallic state of the exsolved Ni by XPS when changing the reducing atmosphere from hydrogen (H_2_) to HArmix (5% H_2_ + 95% Ar). Additionally, a notable difference between non-exsolved and exsolved samples was observed, with non-exsolved samples displaying a nanocrystalline or amorphous homogeneous structure, and the exsolved samples showing a columnar structure and a Ti-poor region. Here we examine the exsolution process of A-site excess STNO thin films more closely.

In this work, we present two types of thin film structures to investigate the effect of different synthesis and processing conditions. Thin films of the first type consist of a single layer (SL) of STNO, with varying thicknesses across samples deposited by pulsed laser deposition (PLD) on Si-wafers. We investigated the influence of pre-exsolution annealing of the thin films at a temperature higher than the deposition temperature, exposure of the samples to different reducing atmospheres, and variations in the exsolution time. The thin films of the second type consist of three layers (TL). Here, both the bottom and top layers are made of Ni-free A-site excess STO. The middle layer, however, is made of STNO, the same material as used in the SL-samples.

## 2. Experimental

For this work, two types of targets were utilized. The first is A-site excess strontium titanate (Sr_1.07_Ti_0.93_O_3±*δ*_, STO), and the second is A-site excess Ni-doped strontium titanate (Sr_1.07_Ti_0.93_Ni_0.07_O_3±*δ*_, STNO). They were synthesized by a solid-state reaction utilizing appropriate amounts of precursors (SrCO_3_ 99%, (1% Ba) Johnson Matthey GmbH, CAS: 1633-05-02; TiO_2_ anatase, Sigma-Aldrich, CAS: 1317-70-0; and Ni(NO_3_)_2_·6H_2_O, 99.999%, Sigma-Aldrich, CAS: 13478-00-7), each of which was weighed to synthesize 2.5 g of powder for each pellet. Each mixture of precursors was ball milled using an agate jar with agate balls for 3 h at 300 rpm in de-ionized water (DI H_2_O). They were dried, calcined at 450 °C, and then ball milled under the same conditions. The powders were mixed with a binder (B60/B709, mixed with ethyl acetate) and pressed into a pellet of 20 mm diameter using a hand press (Atlas Manual Hydraulic Press 15T, Specac). The green bodies were sintered at 1100 °C for 12 h.

The targets were utilized in a pulsed laser deposition setup (PLD, Surface-Tec system, laser: Coherent COMPex Pro 205F, KrF, wavelength: 248 nm), where Si wafers (Si 〈100〉, 2′′, Si-mat) were used as substrates. The substrate temperature was maintained at 600 °C, with a target to substrate distance of 9 cm for all depositions. The chamber was evacuated before deposition, and oxygen gas was used to reach a pressure of 0.01 mbar throughout the process. The target was irradiated with 3.0 J cm^—2^ at a repetition rate of 10 Hz. The SL-samples utilized only the Ni-doped target (STNO), and varying thin film thicknesses were achieved by changing the number of shots. The TL-samples were deposited utilizing both targets. One single wafer was used for deposition, where four different deposition spots were utilized (see the Text in the ESI for more details†). For each film, 10 000 shots were applied to the STO target before the same was repeated for the STNO target, and finalized with another round of 10 000 shots on the STO target for the third and last layer. This resulted in an evenly covered wafer. Subsequently the wafer was broken into smaller pieces. This procedure ensured that the same thin film structure was present in all TL-samples.

To study the effect of pre-annealing on the thin films in an ambient atmosphere before exsolution, some SL-thin films were annealed at 900 °C for 2 h with a heating and cooling rate of 5 °C min^−1^. The others did not undergo any thermal treatment before exsolution. The SL-samples were exsolved in a ProboStat™ (NorECs AS, Norway) under a constant flow of either a H_2_ or HArmix atmosphere. The system was initially flushed with Ar for 15 min at room temperature before switching to the exsolution gas and increasing the temperature to 800 °C with a ramp rate of 5 °C min^−1^ during heating and cooling. While only two different gases were utilized to facilitate exsolution, different exsolution times, *i.e.*, time the samples spent at 800 °C, were utilized, including 30 min and 60 min.

The TL-samples were exsolved in a ProboStat™ under a constant flow of HArmix with a similar procedure to the SL-samples. The exsolution period was the only variable condition for these samples. They were maintained at 800 °C in HArmix for 0 min (immediate transition from heating at 800 °C to cooling), 5 min, 30 min, and 600 min. An overview of all samples, including their names and synthesis conditions is given in Table 1, and an explanation of the naming convention is given in the Text on naming in the ESI.† The preparation method for the different samples for transmission electron microscopy (TEM) is provided in Note 2 on sample preparation in the ESI.†

**Table 1:** The name, thickness, number of pulses, exsolution gas and time, and whether annealing occurred for all samples presented are summarized in the table

Name	Thickness	No of pulses	Exsolution gas	Exsolution time	Annealing	Note
SL-STNO	1.1 μm	20 000	—	—	No	
SL-1.1μm-NA-H5-60	1.1 μm	20 000	HArmix	60 min	No	
SL-1.1μm-A-H5-60	1.1 μm	20 000	HArmix	60 min	Yes	
SL-1.1μm-A-H2-60	1.1 μm	20 000	H_2_	60 min	Yes	
SL-520nm-A-H2-30	520 nm	10 500	H_2_	30 min	Yes	
SL-1.3μm-A-H2-30	1.3 μm	24 000	H_2_	30 min	Yes	
TL-STNO	1 μm	3 × 10 000	—	—	No	
TL-NA-H5-0	1 μm	3 × 10 000	HArmix	0 min	No	
TL-NA-H5-5	1 μm	3 × 10 000	HArmix	5 min	No	
TL-NA-H5-30	1 μm	3 × 10 000	HArmix	30 min	No	
TL-NA-H5-600	1 μm	3 × 10 000	HArmix	600 min	No	
TL-NA-H5-600F	1 μm	3 × 10 000	HArmix	600 min	No	FIB

The samples were primarily analyzed by scanning TEM (STEM) utilizing an FEI Titan G2 60-300 instrument (see Note 3 in the ESI for more details†), along with Energy-Dispersive X-ray Spectroscopy (EDS).

## 3. Results and discussion

**Fig. 1:**
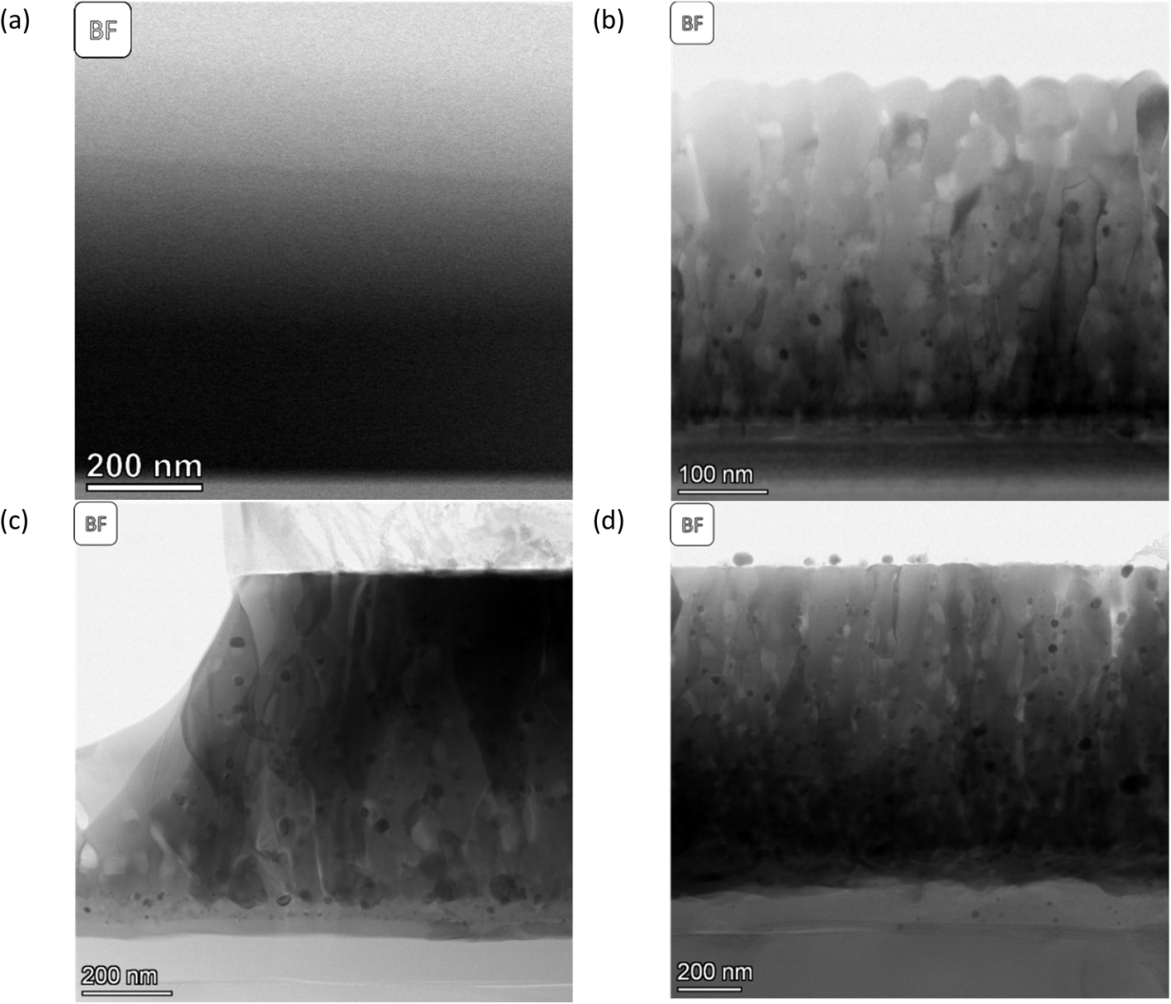
BF-STEM images of (a) SL-STNO, (b) SL-1.1μm-NA-H5-60, (c) SL-1.1μm-A-H5-60, and (d) SL-1.1μm-A-H2-60. Here (c) and (d) have been pre-annealed in an ambient atmosphere at 900 °C for 2 h.

The morphology and structure of the SL-samples can be grouped into two different categories. The as-deposited SL-STNO has a nanocrystalline or amorphous thin film morphology, without any columnar structures, as seen in Fig. 1(a), and emphasized in the XRD data in Fig. S12.† The comparison of the STEM studies of the as-deposited and exsolved samples revealed that after exsolution, irrespective of annealing or exsolution conditions, the thin films exhibit crystalline columnar structures originating from a thin layer at the interface between the thin film and the substrate (see Fig. 1(b)–(d)). The distinct layer at the thin film–substrate interface consists of a Ti-free region, as shown by EDS in Fig. S1–S3.† Despite their structural differences, all thin films presented in Fig. 1 have a similar thickness of 1.1 μm. The atomic weight percentages of Ni, Ti, and Sr in different samples are summarized in Table S1.†

The MNPs created by the exsolution process are visible in Fig. 1(b)–(d). The difference between these samples stems from their synthesis conditions. SL-1.1μm-NA-H5-60, shown in Fig. 1(b), has been exsolved in HArmix for 60 minutes, and shows Ni MNPs with an average radius of approx. 7.8 nm, as shown in Fig. S1.† In contrast, SL-1.1μm-A-H5-60, (shown in Fig. 1(c) and S2†) was annealed prior to exsolution in HArmix for 60 minutes, leading to an average Ni MNP particle size of 12.8 nm. For the SL-1.1μm-A-H2-60 sample, which was annealed and then exsolved in H_2_ for 60 minutes (shown in Fig. 1(d) and S3†), the average Ni MNP particle size is similar, measuring 12.3 nm.

**Fig. 2:**
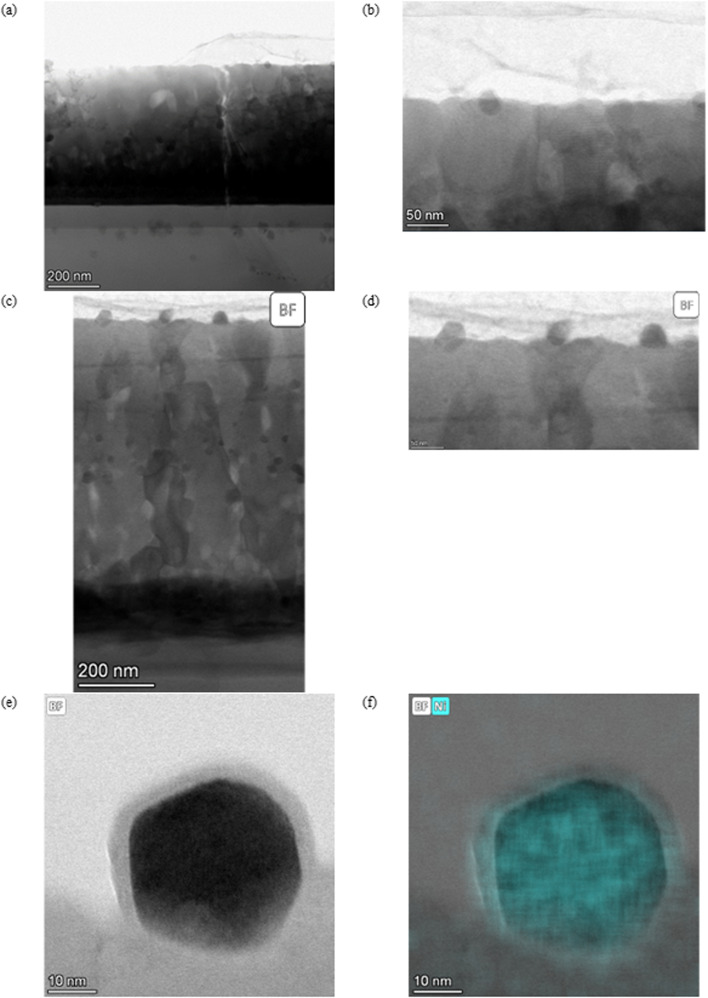
(a) A BF overview image of SL-1.1μm-A-H2-30 and (b) a zoomed in picture of a socketed particle are shown. (c) A BF overview image of SL-520nm-A-H2-30 and (d) a higher magnification image of three socketed particles with varying degrees of socketing. (e) A BF image of a socketed particle in SL-520nm-A-H2-30 and (f) the EDS image indicating Ni. A NiO layer can also be seen on the particle.

**Fig. 3:**
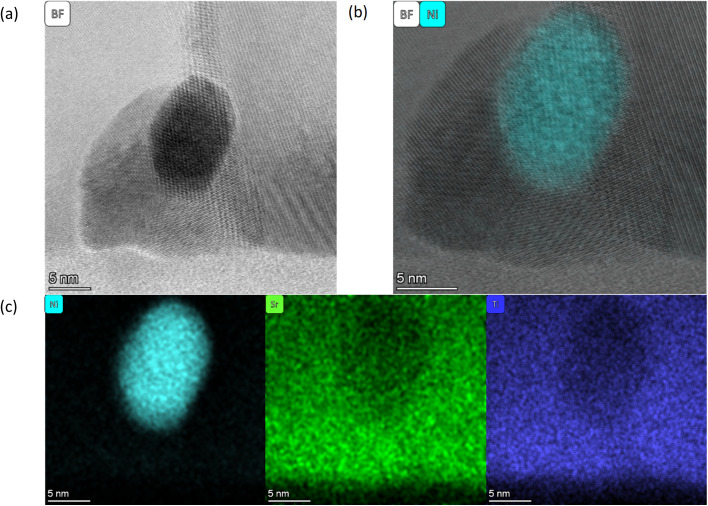
All images are taken from the SL-1.1μm-NA-H5-60 sample shown in Fig. 1(b). (a) a HR BF STEM image of a single particle within the matrix, (b) a HR BF STEM image with the Ni EDS map overlaid, and (c) individual EDS maps for Ni (left), Sr (middle), and Ti (right).

**Fig. 4:**
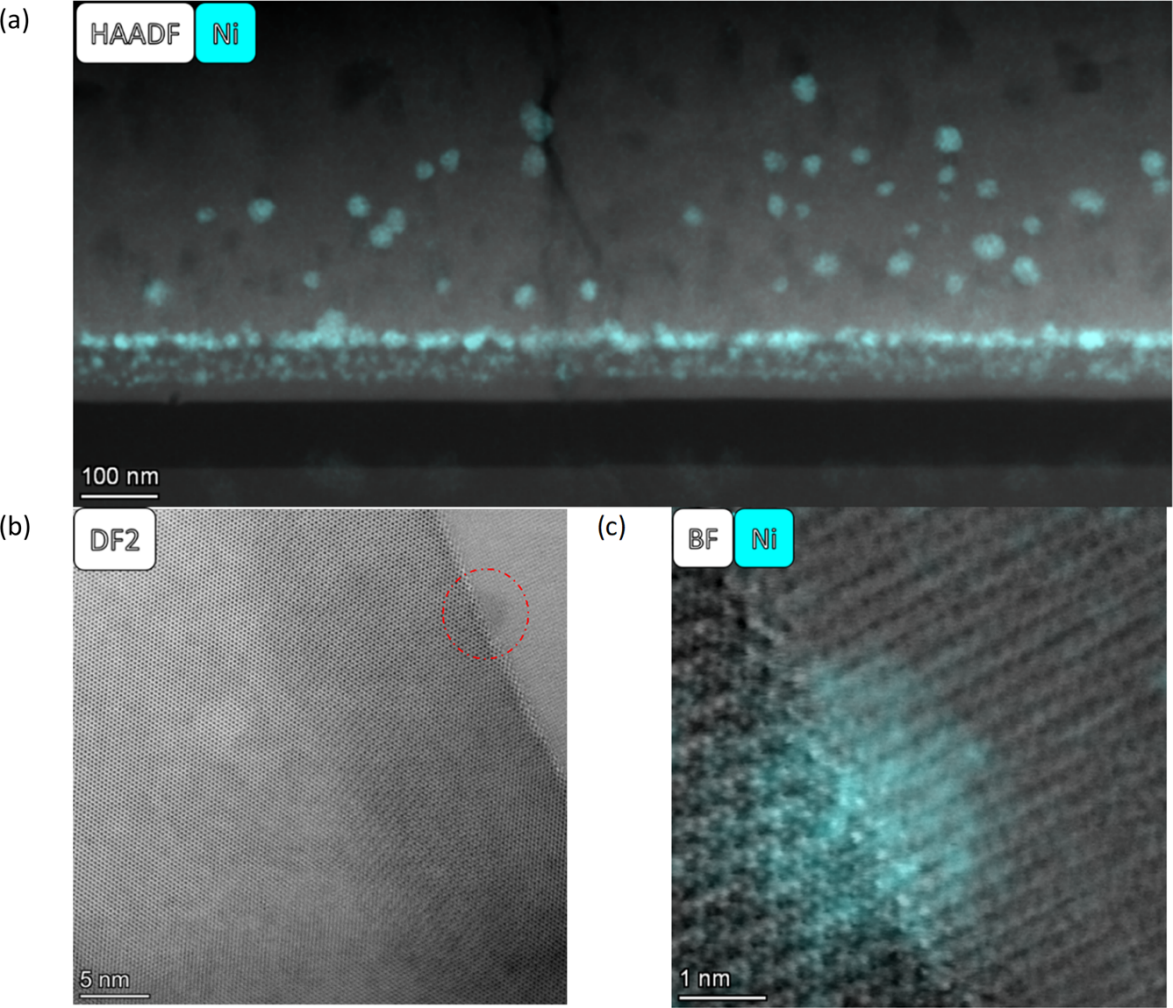
(a) A HAADF image of SL-520nm-A-H2-30 with the Ni EDS map overlaid. (b) HR DF2 STEM image of an interface with a visible particle is displayed (indicated by the red circle). (c) The HR BF STEM image overlaid with the Ni EDS of the particle in (b).

**Fig. 5:**
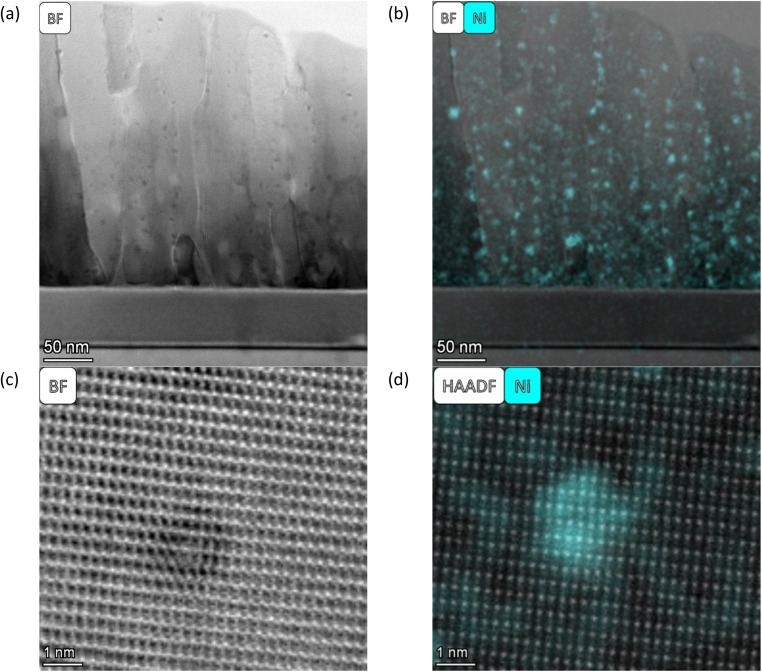
(a) A BF STEM image of SL-1.3μm-A-H2-30 is shown. (b) The BF STEM image overlaid with the Ni EDS map is displayed. (c) The HR BF STEM image of the bulk and (d) the HR HAADF STEM image overlaid with the Ni map.

**Fig. 6:**
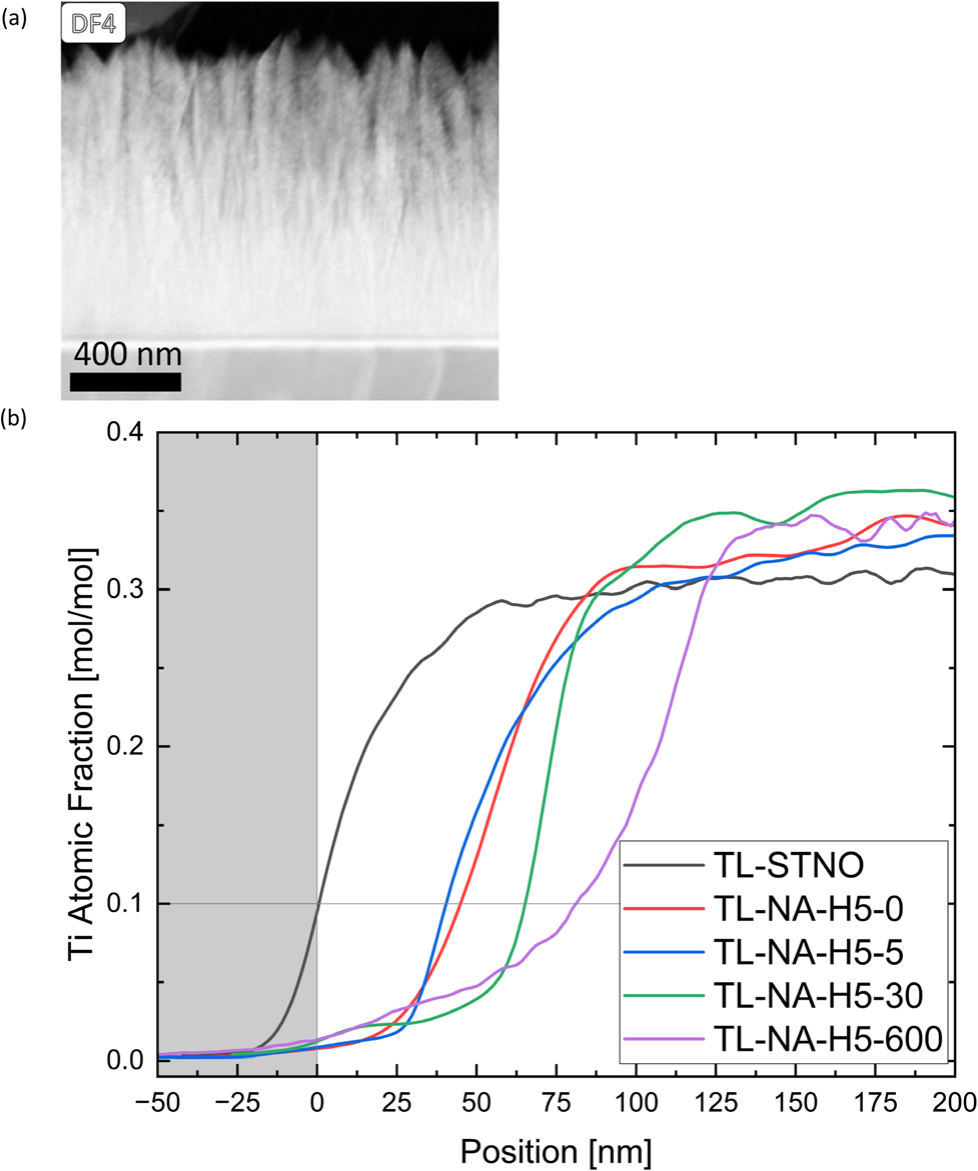
(a) A DF4 STEM image of the TL-STNO sample is shown. (b) The atomic fraction of Ti measured by EDS for all TL samples is plotted against the position. The different samples were aligned at 0 nm at the point where 10% Sr was present.

As described by Kousi *et al.*,^19^ we also observed MNPs within the bulk of all exsolved thin film samples. In fact, we showcase exsolved Ni MNPs socketed to varying degrees on the surface and visible in the bulk (see Fig. 1–6). Particles on the surface can be seen in Fig. S11.† Some of these MNPs are located near internal surfaces, *e.g.*, gaps between columns or grain boundaries, where a higher concentration of defects is present and can act as nucleation sites. An example of such an MNP is shown in Fig. 3(a) and (b) with high-resolution BF-STEM (HR-BF-STEM) and HR-BF-STEM overlaid with EDS. Moreover, we have indicated regions of particles at grain boundaries in Fig. S14.† The grain boundary is visible just to the right of the center of the particle. From the corresponding EDS (see Fig. 3(c)), it is evident that the entire MNP is surrounded by the matrix.

In general, the particles are spatially separated across all samples. SL-520nm-A-H2-30 shows an average particle size of 11.3 nm. Fig. 4(a) shows an overview of the thin film, and Fig. 4(b) and (c) display a particle at a grain boundary, appearing spherical and much smaller than the average particle. SL-1.3μm-A-H2-30 shows the smallest average particle size, with a radius of 2.5 nm. Fig. 5(a) shows a BF STEM image, giving an overview of the thin film structure. Fig. 5(b) shows a BF STEM image with the Ni EDS map of the same region, and Fig. 5(c) and (d) display a Ni-rich region, with no apparent disturbance in the crystal lattice surrounding it, indicating that the particle could be a true bulk exsolution particle.

The influence of the atmosphere during exsolution is negligible in contrast to the influence of pre-annealing. In this study, annealing led to far larger particles, with the average radius of particles being up to one and a half times bigger in annealed samples than the average radius in samples that have otherwise been treated similarly. The thickness of the samples influences the particle size, such that thicker films either have smaller average particle sizes or need to be exsolved for longer duration to achieve the same particle size as in thinner films. Generally, the homogeneity of exsolution and the size of the MNPs in these A-site excess thin films fall somewhere between those of A-site stoichiometric and A-site deficient films, as seen in, *e.g.*, ref. 9 and 24. We theorize that this is due to the layered structure of the A-site excess STO, enabling better oxide and cation transport, supporting exsolution, compared to the A-site stoichiometric material, but worse than the A-site deficient one.

At the thin film–substrate interface, a distinct Ti-free region is noticeable, with a Si signal present, as evident in Fig. S1–S3.† Moreover, there is only a miniscule Ni-signal in this region, indicating that the Sr might have diffused into SiO_2_ rather than Si diffusing into the film. To study the movement of different ions within the samples, the TL samples were studied. The influence of time on the exsolution process was observed, specifically regarding the location of Ni, and whether the Ti-free region grows over time. The presence of columnar structures in the non-exsolved TL-STNO sample (see Fig. 6(a)) contrasts with our previous observation of nanocrystalline/amorphous thin films in the SL samples and in our previous study.^18^ However, the Ti-free region is not present in the as-deposited region, as shown in Fig. S6.†

**Fig. 7:**
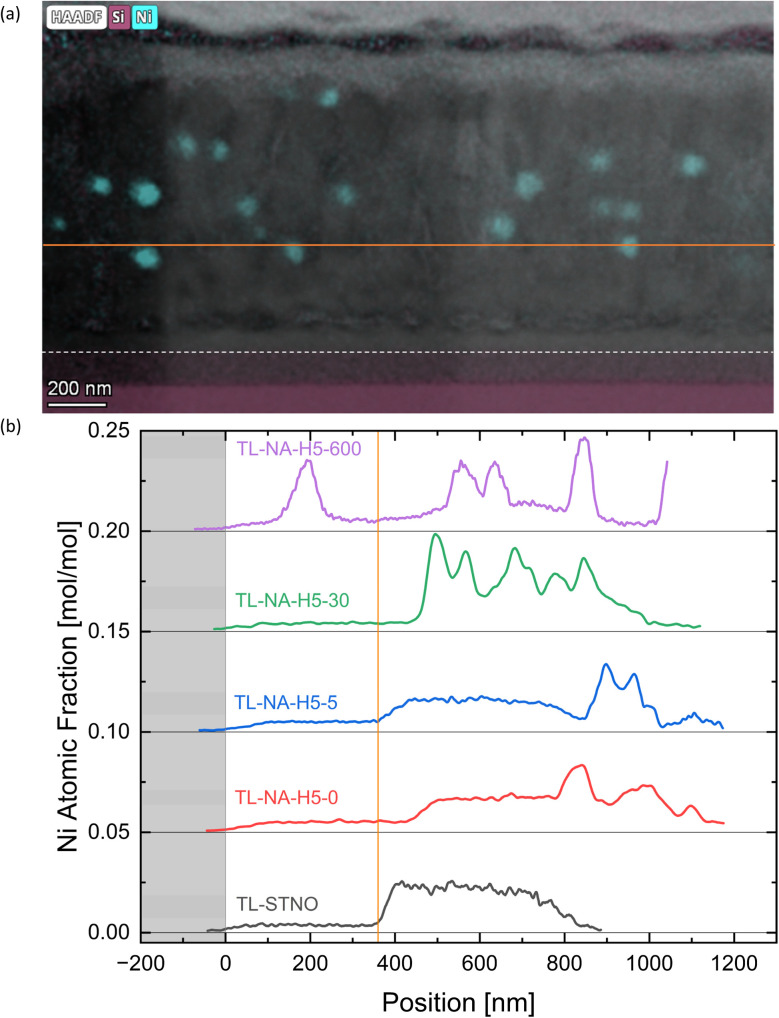
(a) The HAADF image overlaid with the Ni and Si EDS map of TL-NA-H5-600F is shown. (b) The atomic fraction of Ni plotted against the position in the sample. 0 nm marks where all samples have a 10% atomic fraction of Ti, and the orange line marks the onset of Ni in TL-STNO. Similar lines are shown in (a).

In Fig. 6(b), the EDS-measured concentration of Ti is plotted as a function of position for TL-STNO, TL-NA-H5-0, TL-NA-H5-5, TL-NA-H5-30, and TL-NA-H5-600. The corresponding EDS maps can be found in Fig. S6–S10.† The Ti-free region grows over time, reaching roughly 80 nm after 600 min. It is worth noting that the Si signal is not negligible in the Ti-free region, suggesting the presence of a Si–Sr–O phase. Finally, the influence of time on the position and size of Ni can be seen in Fig. 7; the TL-NA-H5-600F sample is shown. In Fig. 6(a), the HAADF-STEM image is overlaid with the EDS signal of Si and Ni, providing an overview. The two layers on top of the thin film are carbon and tungsten from the FIB process. The white dashed line indicates 10% atomic fraction of Ti, and the orange line indicates the onset of the Ni signal in TL-STNO. The Ni MNPs primarily remain concentrated in the originally Ni-doped region, but can also be found beyond it, especially towards the surface, but for longer annealing times (600 min), the MNPs are also observed towards the substrate and are larger than those in any other sample.

In Fig. 7(b), the Ni-signal in each TL sample is plotted with Ti as a reference point. The samples were aligned so that 10% of the atomic fraction of Ti is located at 0 nm, and the signal was taken from the entire images shown in Fig. S6–S10.† The graph shows the diffusion of Ni, with peaks generally associated to the Ni-MNPs in bulk. The purpose of the FIB sample in Fig. 7(a) was to ensure that the particles move even if no grinding is involved in the preparation of the sample. The lines in Fig. 7(a) correspond to the lines in Fig. 7(b) with the white line near the thin film–substrate interface indicating the 0 nm position.

## 4. Conclusion

In this study we have demonstrated the influence of thin film thickness, time, atmosphere, and pre-annealing on the result of exsolution. In contrast to Weber *et al.*^22^ we have not observed any Ni-rich secondary phases in the as-deposited thin films. The results show the presence of spherical or ellipsoidal particles in all samples, with the pre-annealing step leading to larger particles in general. In contrast to Syed *et al.*,^21^ no particles with shells or neighboring particles of a different element were observed. The influence of the atmosphere is minimal, meaning that both HArmix and H_2_ lead to similar particle sizes, with exsolution occurring throughout the thin films, including both the surface and bulk. While most particles are located near grain boundaries or other extended defects, as indicated in Fig. S14,† there are some that appear within an undisturbed crystal structure, indicating that their presence did not cause any defects either. Time and thin film thickness have opposite effects, as longer time periods can be used to achieve the same average radius of particles in thicker films.

Finally, an interfacial region is formed during exsolution, where a Ti-poor Si–Sr–O region forms that grows over time. For pre-annealed samples, the interface between the Si–Sr–O region and STO has an increased Ni content, potentially useful in creating regions of high particle concentration. The ability to control the sizes of nanoparticles through various parameters, *e.g.*, time, film thickness, or pre-annealing, and the potential to create regions of high particle density could be useful for designing devices utilizing exsolution.

## Author contributions

Kevin Gregor Both, synthesized the samples, designed, and performed the processing step, performed parts of the TEM measurements, analyzed the data, and wrote the original draft. Dragos Neagu, Øystein Prytz, Truls Norby, and Athanasios Chatzitakis conceived and supervised the study, and did review and editing.

## Conflicts of interest

There are no conflicts to declare.

## Supplementary Material

NA-006-D4NA00213J-s001

## Data Availability

The data shown in the figures of the article and the corresponding ESI† file are available on request from the corresponding authors.
