# Supplementary material for: Exsolution of Ni nanoparticles in A-site excess STO films
Source: Nanoscale Adv. 2024 Oct 14;6(24):6336–43. doi: 10.1039/d4na00213j (PMC11515931; doi:10.1039/d4na00213j)
Supplement: NA-006-D4NA00213J-s001 [file NA-006-D4NA00213J-s001.pdf]

Supplementary Information (SI) for Nanoscale Advances.
This journal is © The Royal Society of Chemistry 2024

Supplementary Information (SI)

Exsolution of Ni Nanoparticles in A-site Excess STO Films

Authors

Kevin G. Both,[1*] Dragos Neagu,[2] Øystein Prytz,[1] Truls Norby,[3] Athanasios Chatzitakis,[3*]

Affiliations

(1) Centre for Materials Science and Nanotechnology, Department of Physics, University of Oslo, Gaustadalléen 21, NO-0349 Oslo, Norway

(2) Department of Chemical and Process Engineering, University of Strathclyde, 75 Montrose St, G1 1XJ, Glasgow, United Kingdom

(3) Centre for Materials Science and Nanotechnology, Department of Chemistry, University of Oslo, Gaustadalléen 21, NO-0349 Oslo, Norway

*Corresponding authors: k.g.both@smn.uio.no, athanasios.chatzitakis@smn.uio.no

1. Supplementary Tables

Table S1: The EDS atomic% of Ni, Ti, Sr, as well as the ratio of Ni/(Ti+Ni) and stoichiometry of selected films are given in the table. If no regions are indicated, the measurements are of the entire thin film. Regions are marked with a number and the corresponding figure is given in the table. The stoichiometry of three regions were not resolved due to the large presence of Si. The intended (nominal) stoichiometry is also given (based on the targets used).

Film	Ni (at%)	Ti (at%)	Sr (at%)	Sr/(Ti+Ni)	Stoichiometry	Nominal Stoichiometry	Figure
SL-STNO	3.37	35.03	43.47	1.13	$Sr_{1.13}Ti_{0.91}Ni_{0.09}O_{3.04}$	$Sr_{1.07}Ti_{0.93}Ni_{0.07}O_3$	S13
SL-1.1µm-A-H5-60	2.82	31.69	36.50	1.05	$Sr_{1.05}Ti_{0.92}Ni_{0.09}O_{2.97}$		S2
SL-1.1µm-A-H2-60 Region 1	2.58	32.77	35.33	1	$SrTi_{0.93}Ni_{0.09}O_{2.92}$		S3 (red)
SL-1.1µm-A-H2-60 Region 2	1.02	13.97	31.91	2.12	$Sr_xSi_yO_z$ + $Sr_aTi_b Ni_cO_d$		S3 (yellow)
SL-520nm-A-H2-30 Region 1	1.79	30.78	34.02	1.04	$Sr_{1.04}Ti_{0.95}Ni_{0.05}O_{2.99}$		S4 (yellow)
SL-520nm-A-H2-30 Region 2	5.9	14.88	36.39	01.75	$Sr_xSi_yO_z$ + $Sr_aTi_b Ni_cO_d$		S4 (red)
SL-1.3µm-A-H2-30 Region 1	1.80	21.67	23.80	1.01	$Sr_{1.01}Ti_{0.92}Ni_{0.08}O_{2.94}$		S5 (red)
SL-1.3µm-A-H2-30 Region 2	0.08	0.72	22.51	28.14	$Sr_xSi_yO_z$ + $Sr_aTi_b Ni_cO_d$		S5 (yellow)
TL-STNO Region 1	2.01	26.42	31.10	1.09	$Sr_{1.09}Ti_{0.93}Ni_{0.07}O_{3.02}$	$Sr_{1.07}Ti_{0.93}Ni_{0.07}O_3$	S6 (red)
TL-STNO Region 2	0.00	32.09	37.43	1.17	$Sr_{1.17}TiO_{3.17}$	$Sr_{1.07}TiO_{3.07}$	S6 (yellow)

2. Supplementary Figures

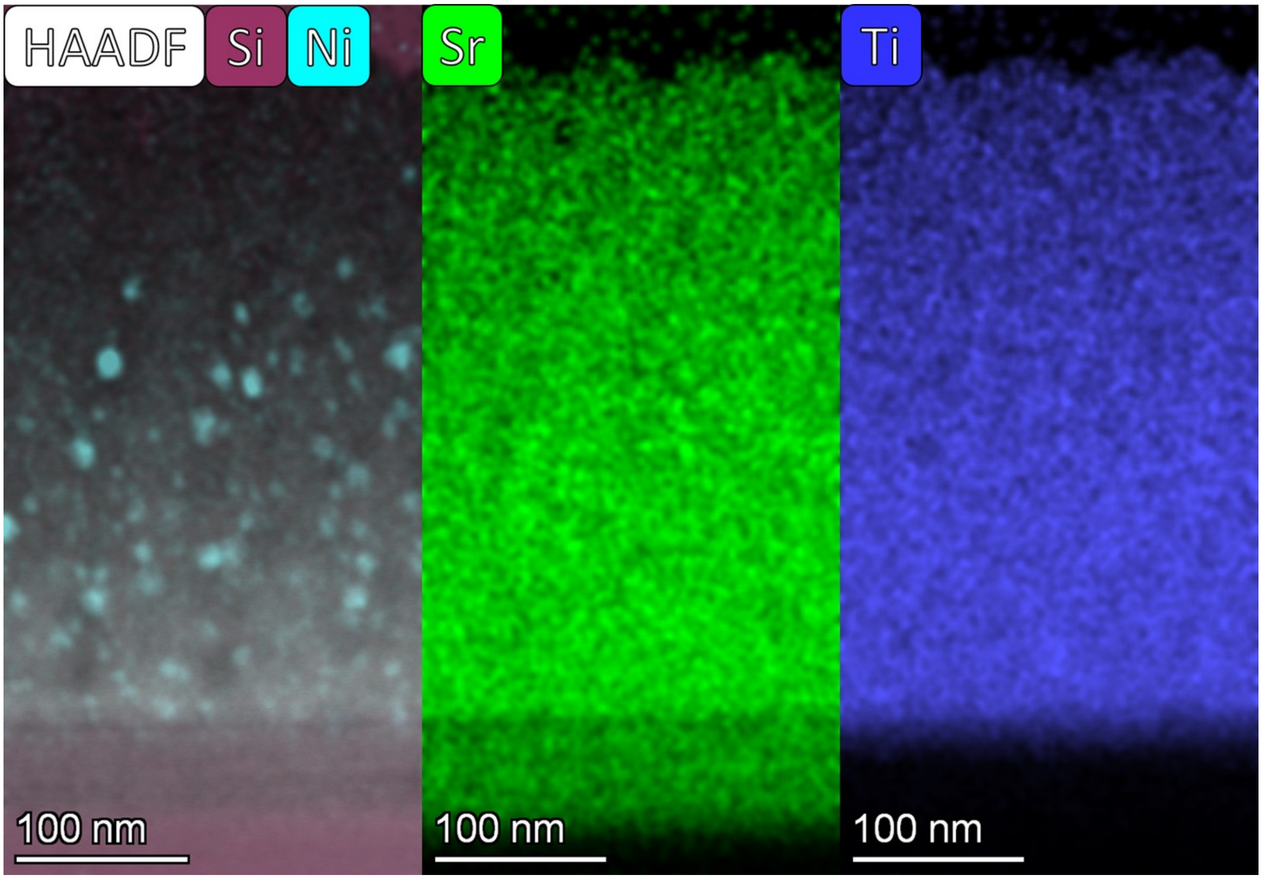

Figure S1: Images of the SL-1.1µm-NA-H5-60 thin film, where (left) is the HAADF STEM image with Si and Ni EDS overlay, (middle) is the Sr EDS map, and (right) is the Ti EDS map.

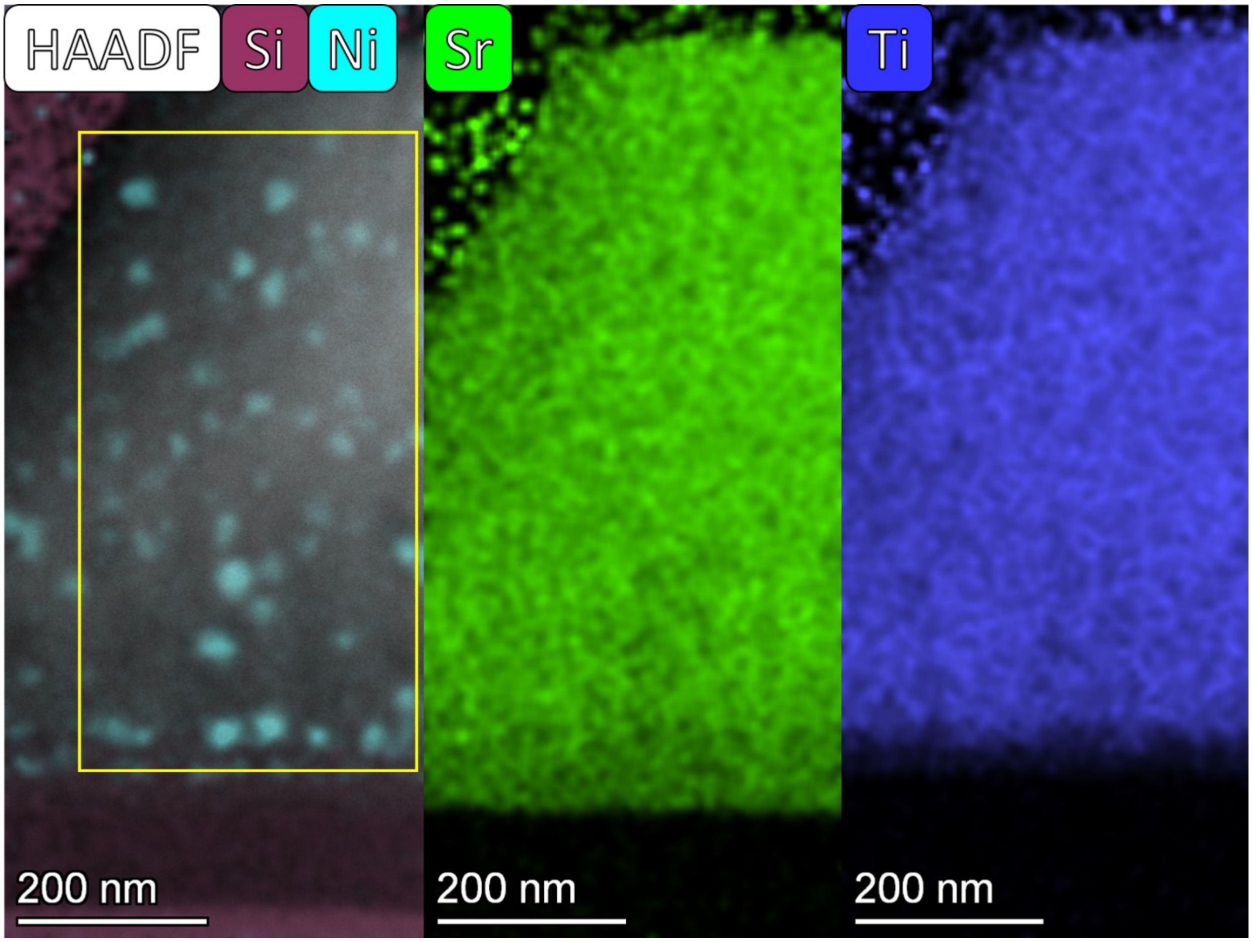

Figure S2: Images of the SL-1.1µm-A-H5-60 thin film, where (left) is the HAADF STEM image with Si and Ni EDS overlay, (middle) is the Sr EDS map, and (right) is the Ti EDS map. The yellow area marks the origin of the EDS data for Table S1.

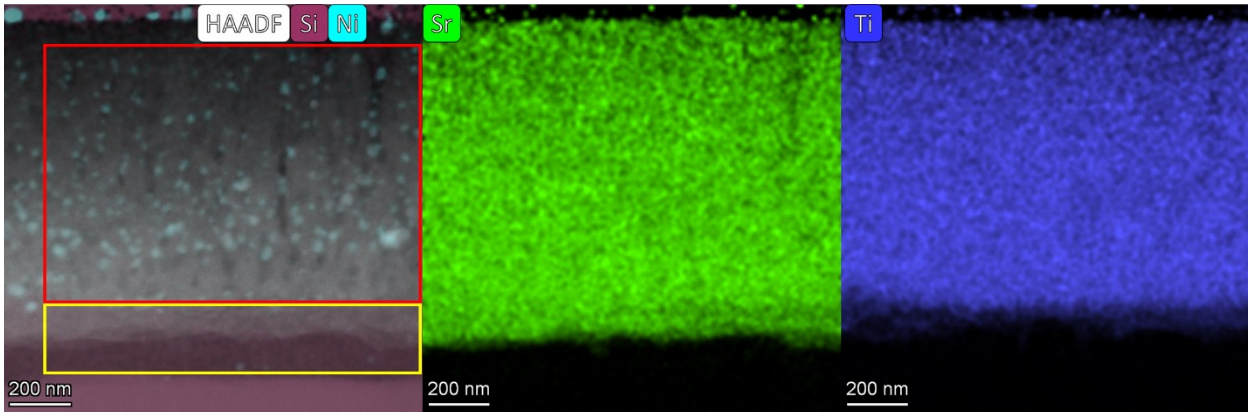

Figure S3: Images of the SL-1.1µm-A-H2-60 thin film, where (left) is the HAADF STEM image with Si and Ni EDS overlay, (middle) is the Sr EDS map, and (right) is the Ti EDS map. The yellow and red areas mark the origin of the EDS data for Table S1.

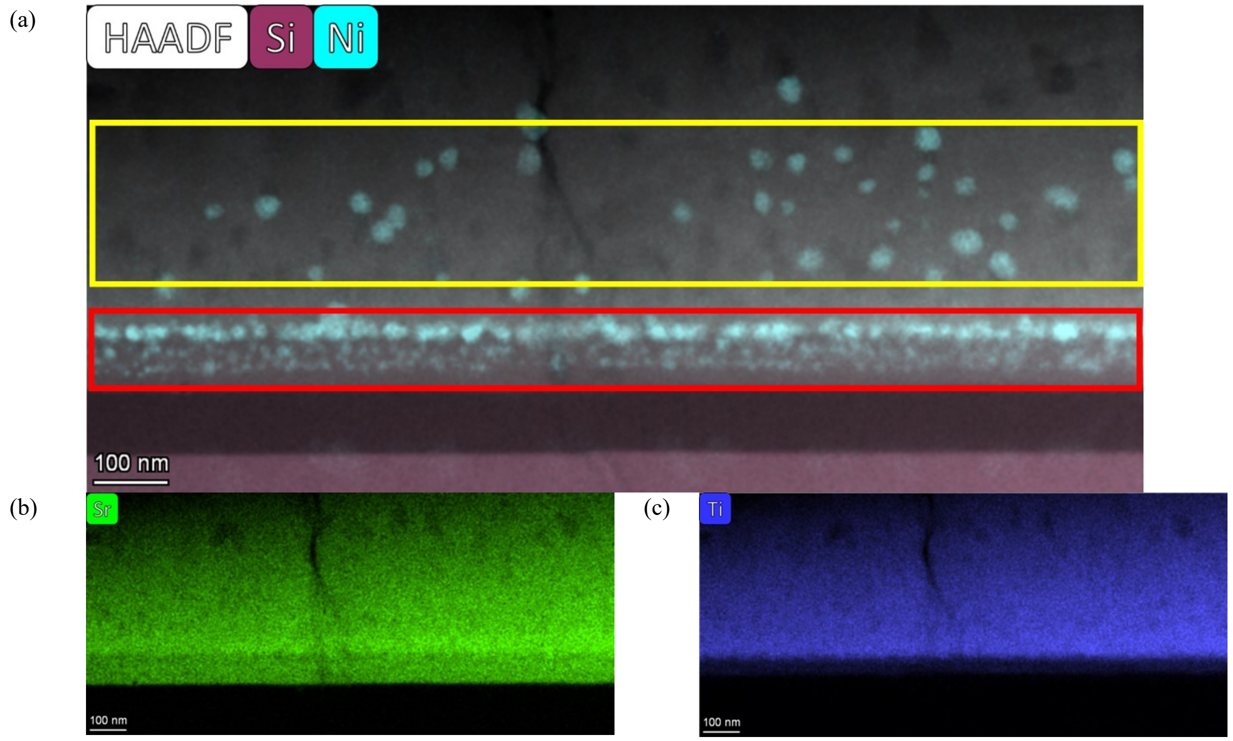

Figure S4: Images of the SL-520nm-A-H2-30 thin film, where (a) is the HAADF STEM image with Si and Ni EDS overlay, (b) is the Sr EDS map, and (c) is the Ti EDS map. The yellow and red areas mark the origin of the EDS data for Table S1.

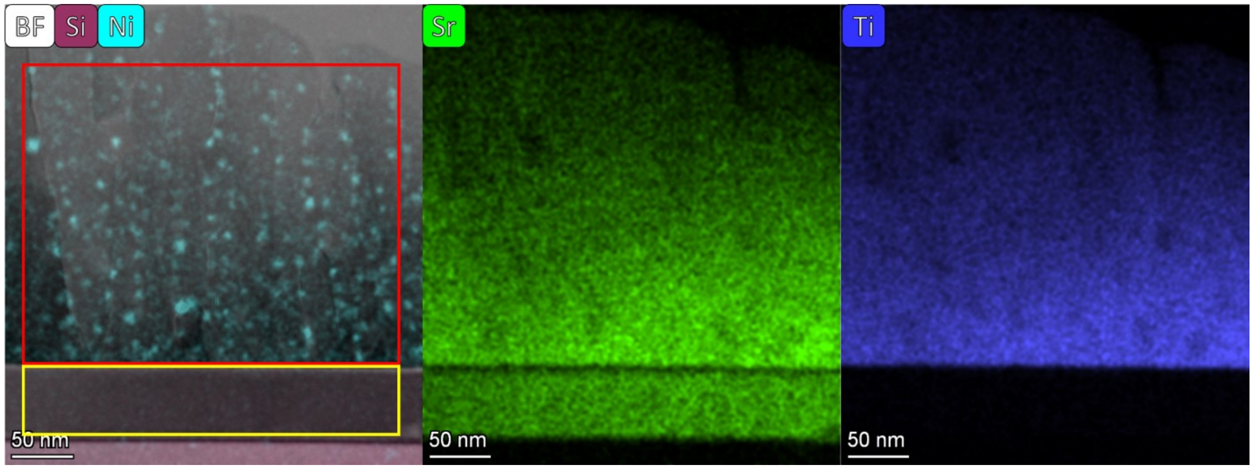

Figure S5: Images of the SL-1.3µm-A-H2-30 thin film, where (left) is the HAADF STEM image with Si and Ni EDS overlay, (middle) is the Sr EDS map, and (right) is the Ti EDS map. The yellow and red areas mark the origin of the EDS data for Table S1.

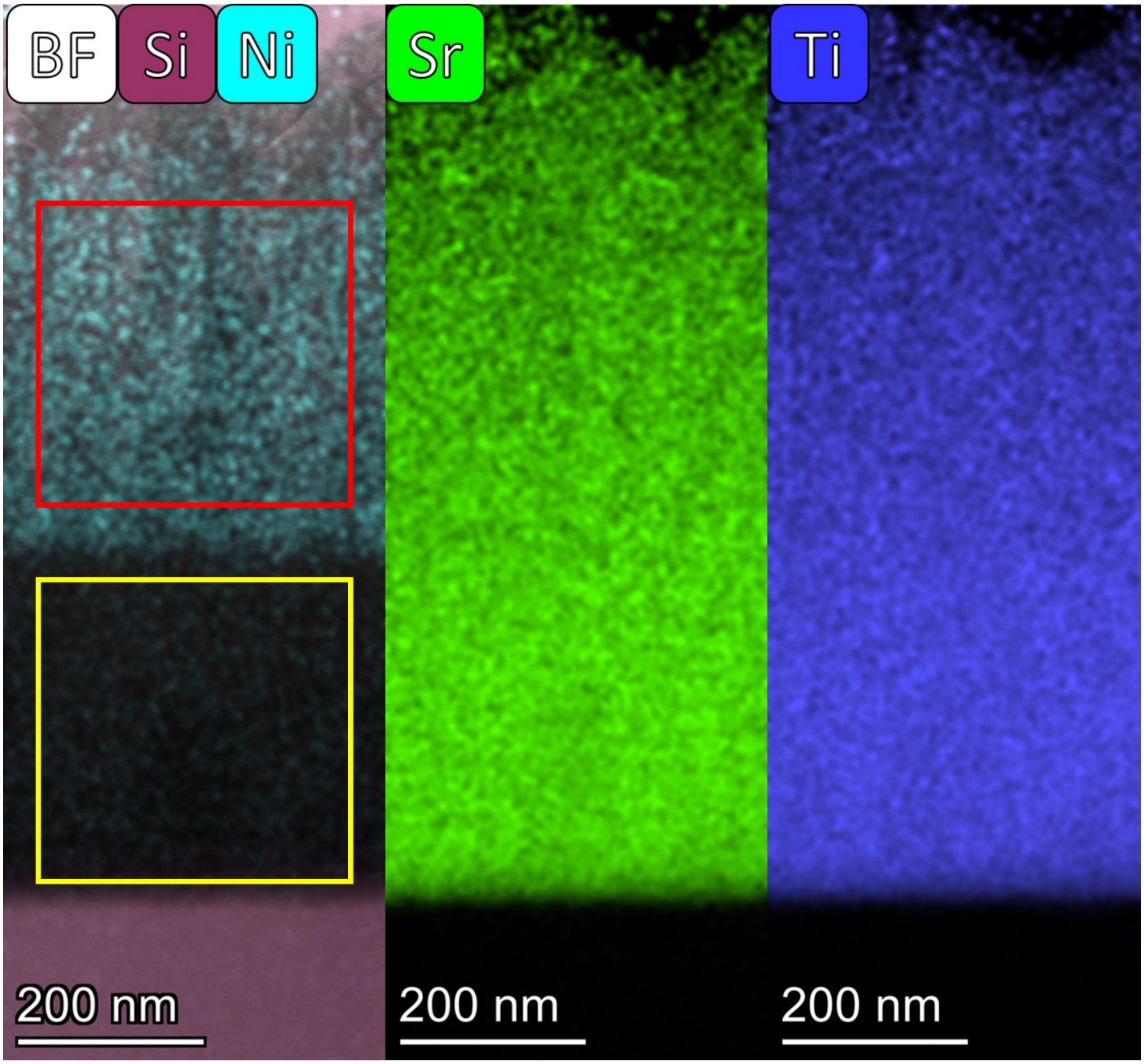

35 Figure S6: Images of the TL-STNO three layer thin film, including (left) a BF STEM image overlaid with Si and Ni EDX maps,
36 (middle) a Sr EDS map, and (right) a Ti EDS map. The yellow and red areas mark the origin of the EDS data for Table S1.

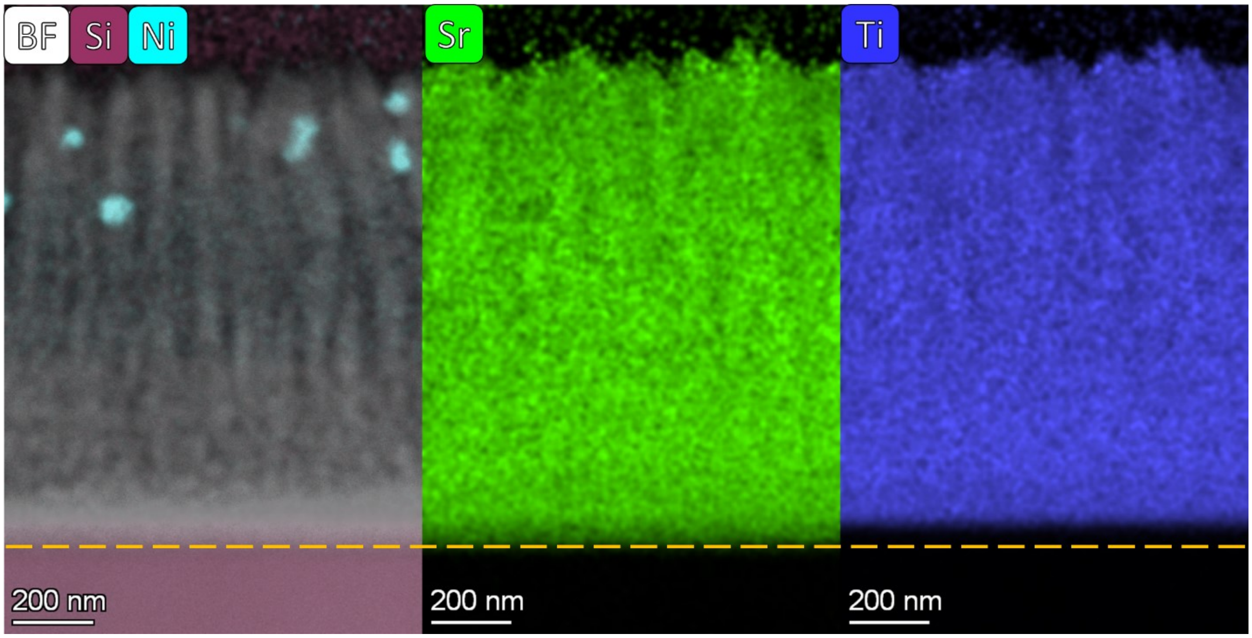

Figure S7: Images of the three layer TL-NA-H5-0, including (left) a BF STEM image overlaid with Si and Ni EDX maps, (middle) a Sr EDS map, and (right) a Ti EDS map. The orange line marks approximately the beginning of the Sr.

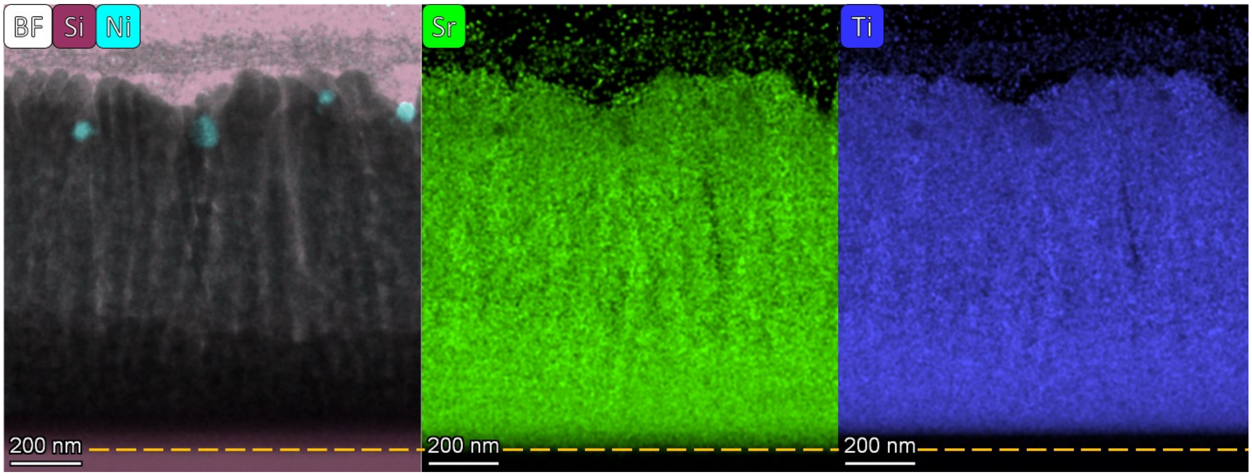

Figure S8: Images of the three layer TL-NA-H5-5, including (left) a BF STEM image overlaid with Si and Ni EDX maps, (middle) a Sr EDS map, and (right) a Ti EDS map. The orange line marks approximately the beginning of the Sr.

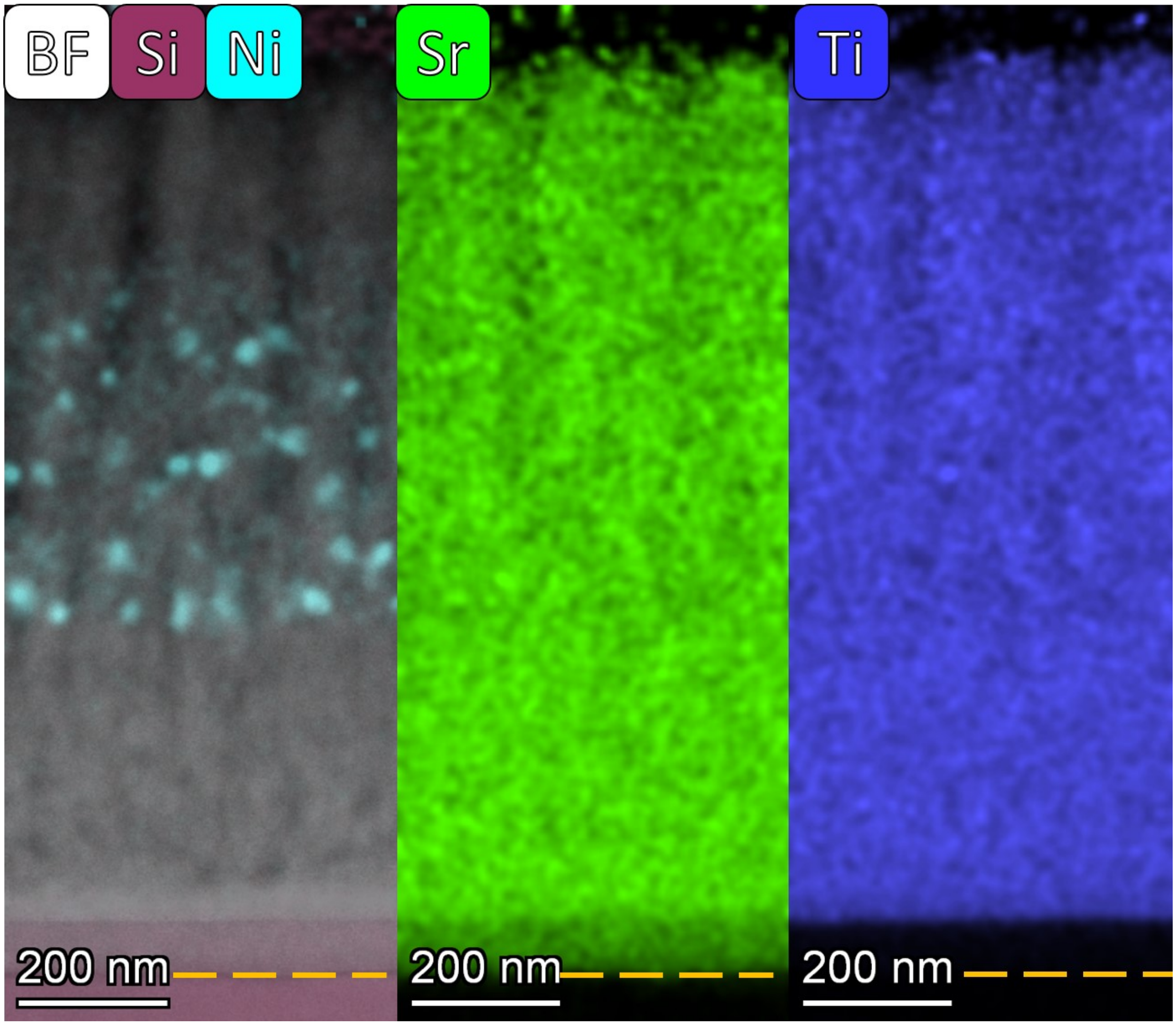

Figure S9: Images of the three layer TL-NA-H5-30, including (left) a BF STEM image overlaid with Si and Ni EDX maps, (middle) a Sr EDS map, and (right) a Ti EDS map. The orange line marks approximately the beginning of the Sr.

(a)

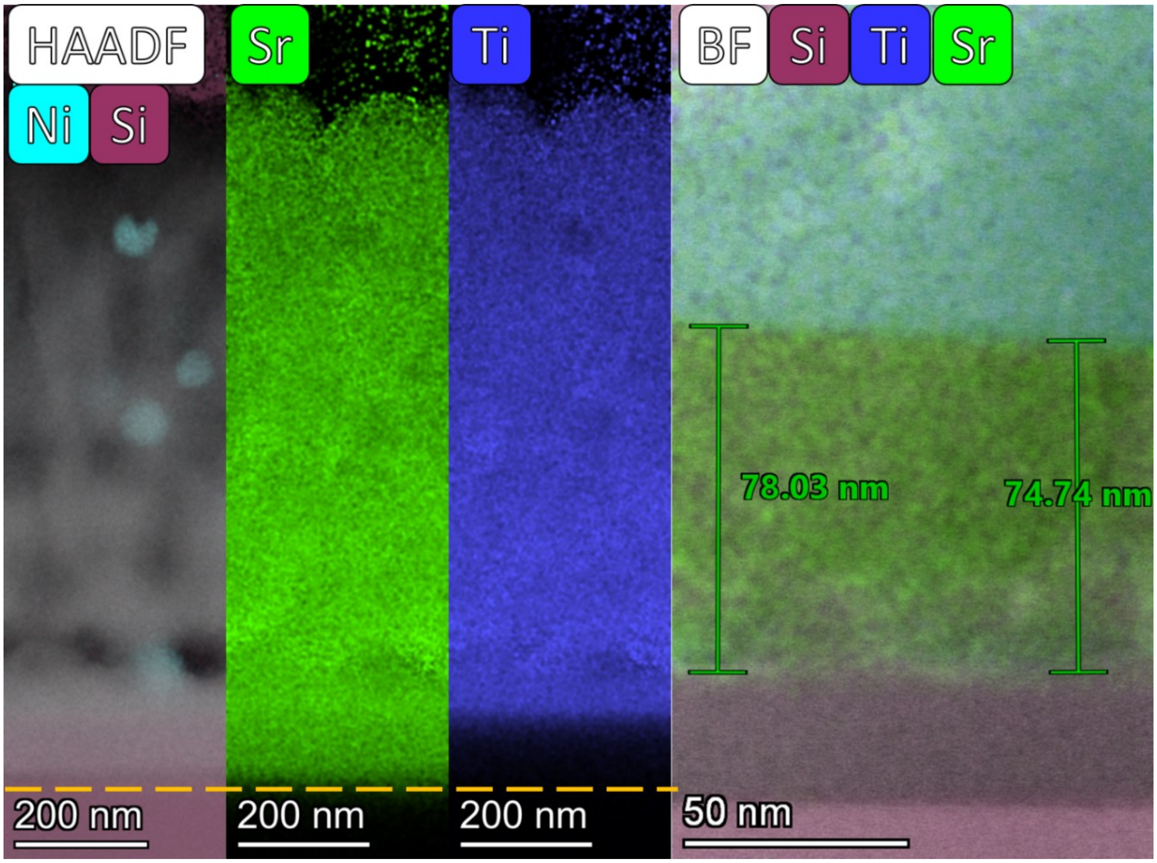
HAADF
Ni
Si
Sr
Ti
BF
Si
Ti
Sr
78.03 nm
74.74 nm
200 nm
200 nm
200 nm
50 nm

(b)

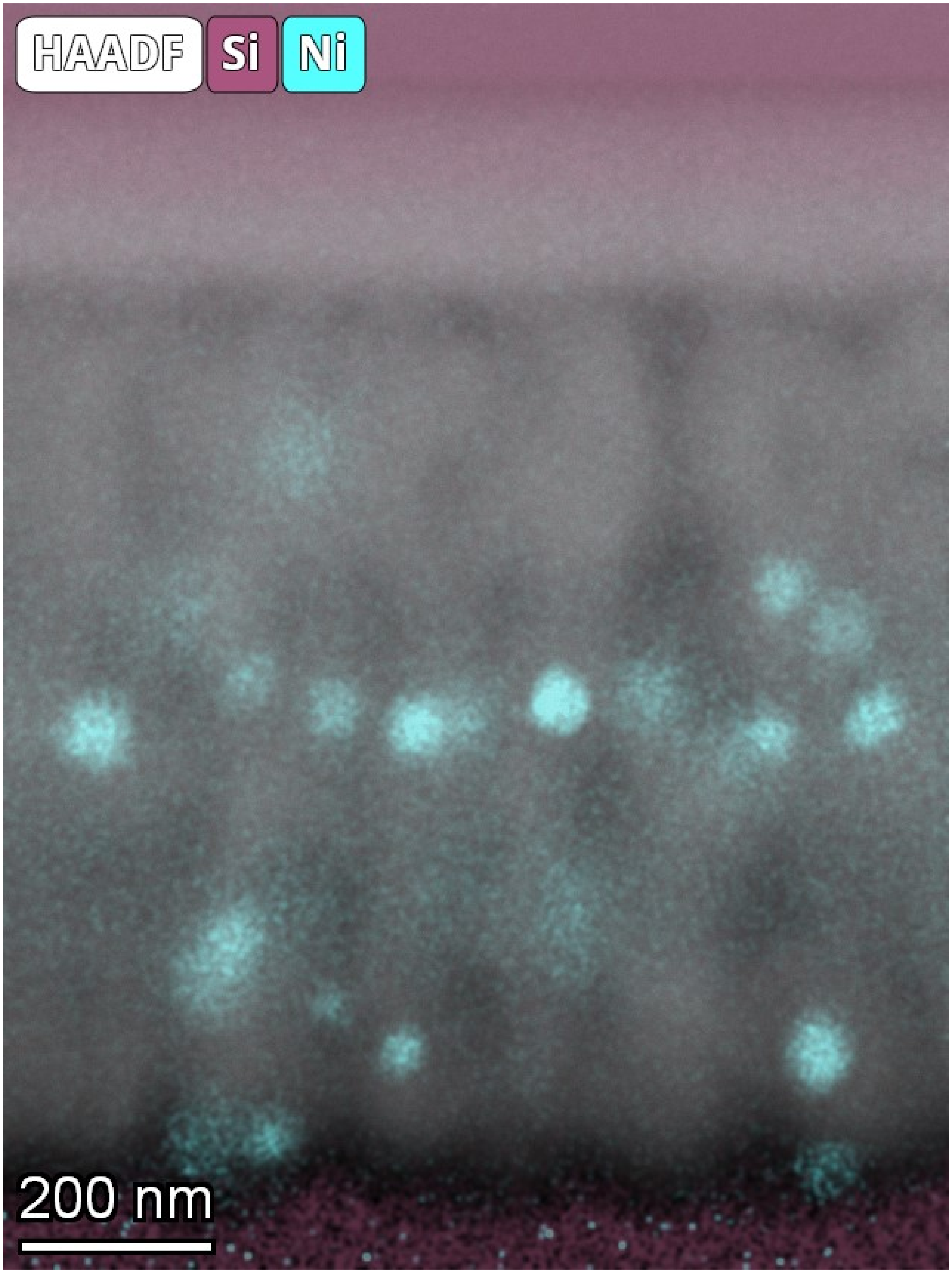

Figure S10: (a) Images of the three layer TL-NA-H5-600, including (left) a HAADF STEM image overlaid with Si and Ni EDX maps, (second from the left) a Sr EDS map, and (second from the right) a Ti EDS map. The orange line marks approximately the beginning of the Sr. (Right) A BF STEM image and the elemental EDS maps for Si, Ti, and Sr. Two measurements for the thickness of the Ti-free layer. (b) The area used to extract the Ni data for Fig. 6 (b).

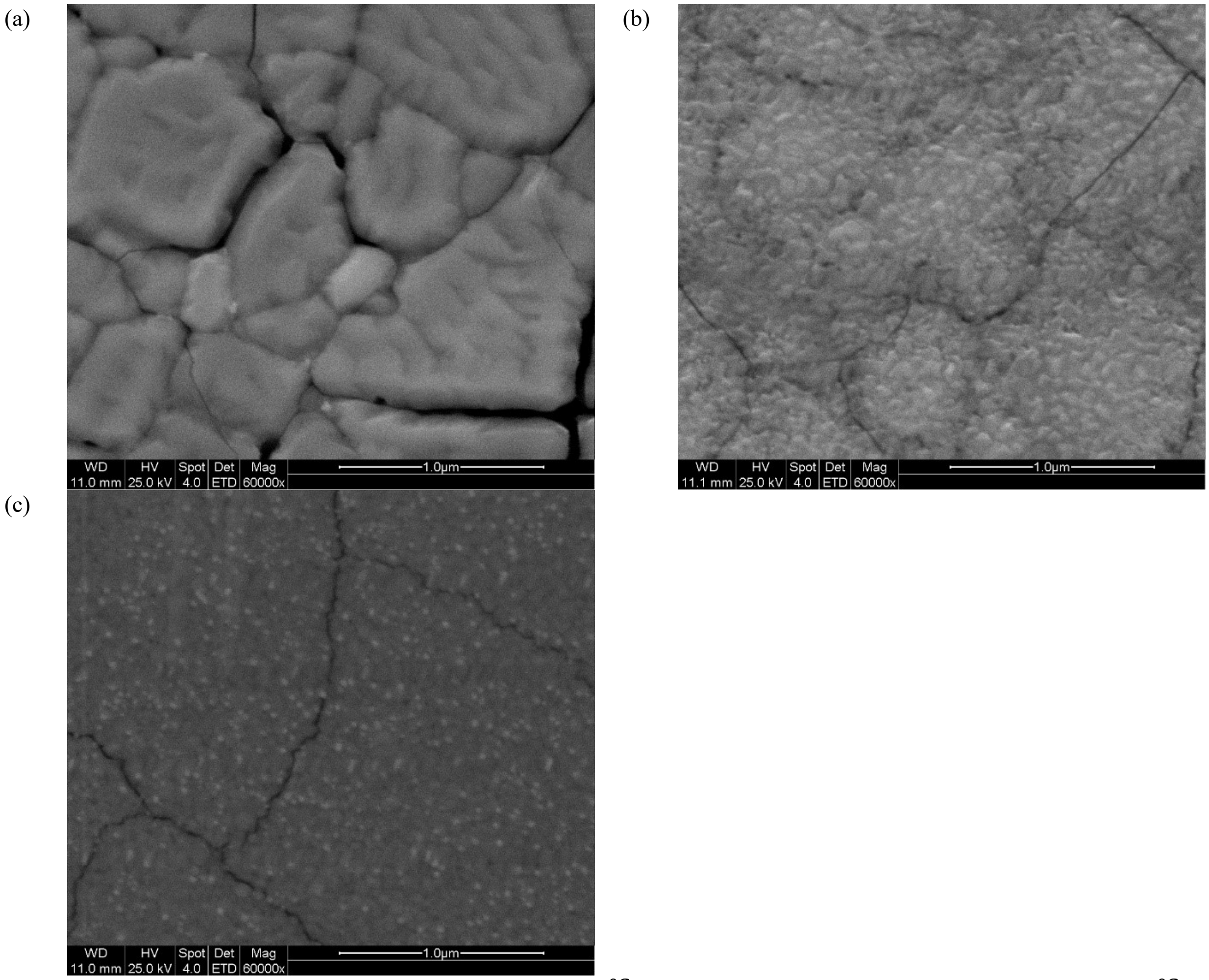

Figure S11: SEM images of the STNO thin film heated to 800 ℃ for 60 min in ambient atmosphere (a), heated to 650 ℃ and immediately quenched in HArmix (b), and of SL-1.1µm-A-H5-60 (c).

Upon reaching the target temperature with the sample heated to 650 ℃ in HArmix (Fig. S11 (b)), the ProboStat™ was removed from the furnace, i.e., the sample was “quenched” in HArmix. This was an attempt to freeze the structure obtained at 650 ℃ in place, and to discover how the structure would change during the heating stage of our synthesis, without introducing a prolonged heating stage.

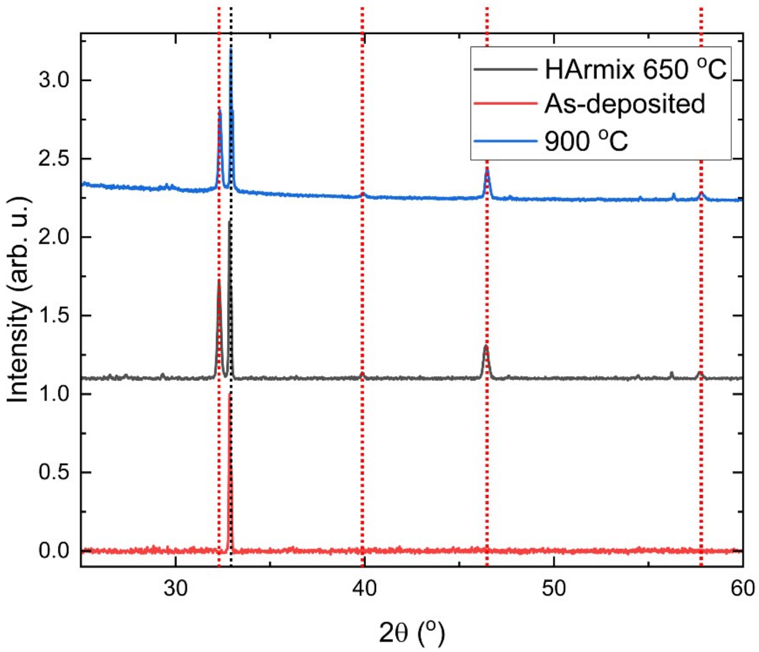

Figure S12: XRD plots of an as deposited sample (red), the same sample heated to 650 ℃ (black) in HArmix, and the same thin film heated to 900 ℃ (blue) in air. The dashed lines correspond to the substrate (Si (substrate, black dashed, PDF 01-078-6300)) and SrTiO3 (red dashed, PDF 01-070-8508), respectively.

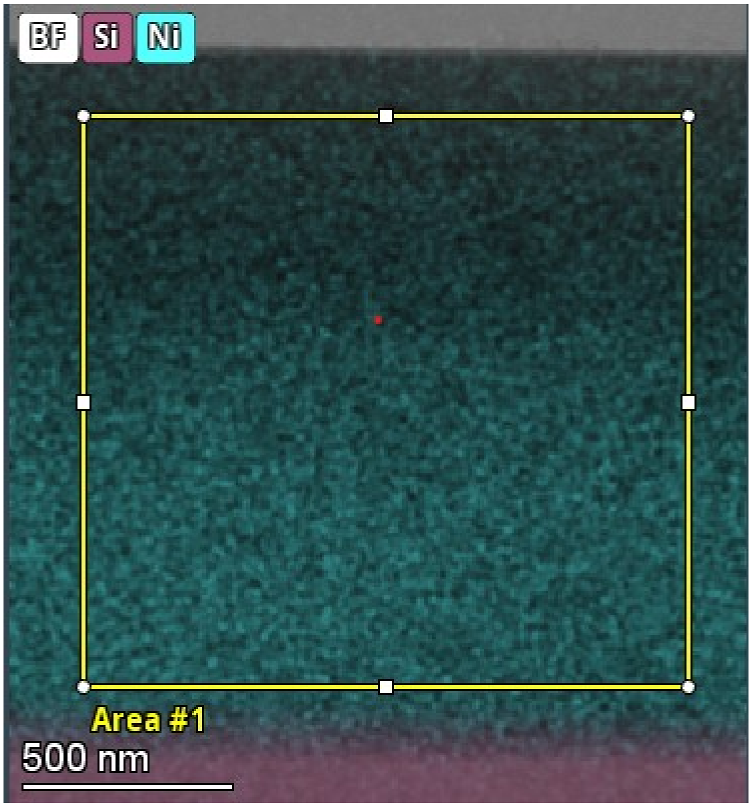

Figure S13: EDS overlay of a STO(Ni) film with Ni and Si colored. The area corresponds to the region where the EDS atomic% was extracted for table S1.

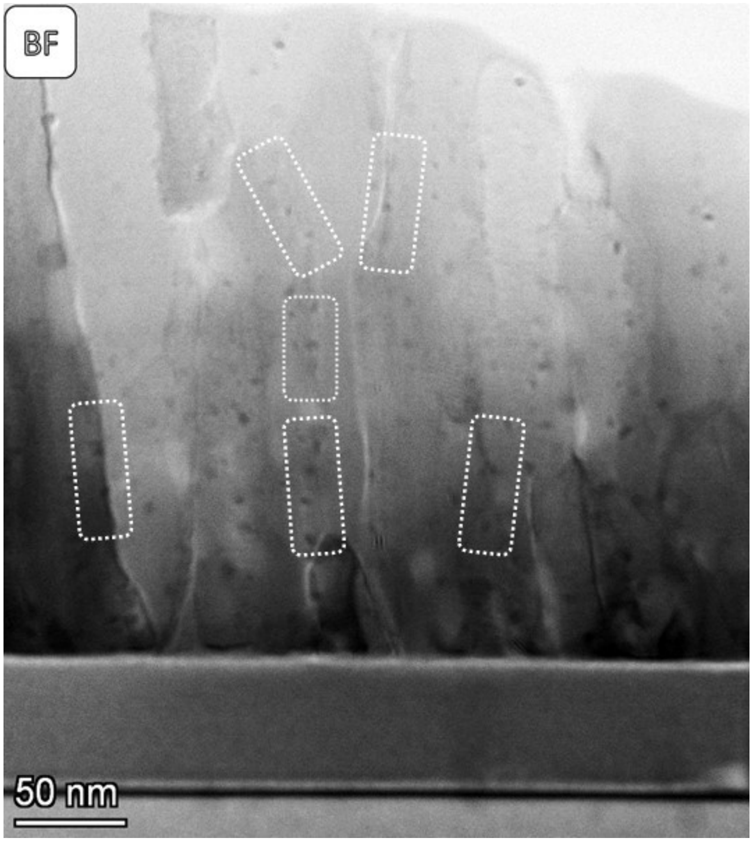

62 Fig. S. 14: The BF STEM image presented in 4 (a), but with white dashed boxes indicating particles at grain boundaries.

(a) (b) (c) (d)

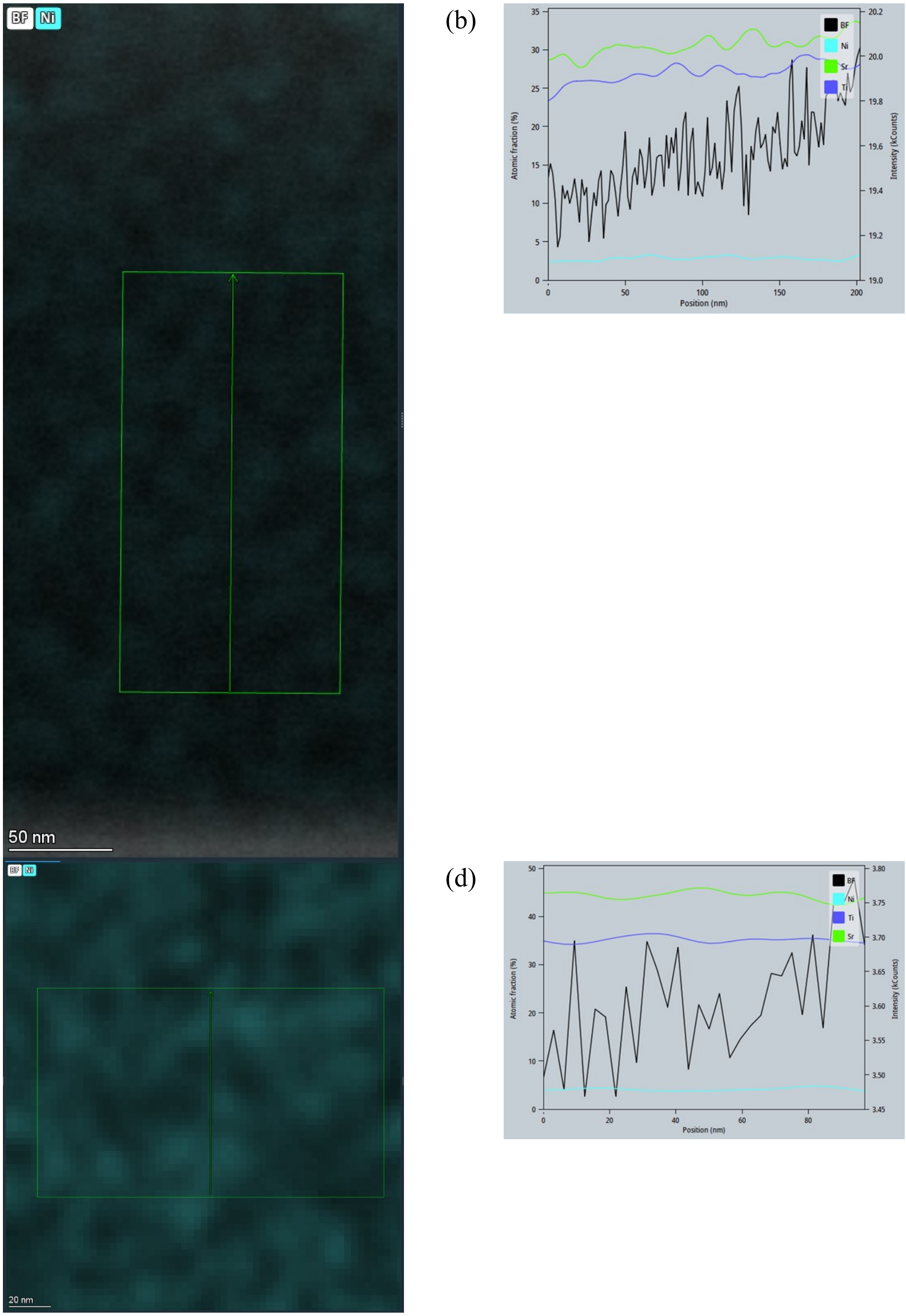

Fig. S15: (a) An area of STNO thin film is shown with (b) the corresponding EDS signal. (c) A different area of the STNO thin film is shown, again with (d) showing the corresponding EDS signal.

3. Supplementary Text

Supplementary Note 1:

The PLD of the three layer samples was achieved by depositing on an entire 2” wafer. The wafer was mounted in the PLD on the sample holder shown in Fig. S15, with deposition position I-IV. These positions label the geometric alignment where the target is directly underneath the substrate, creating the ideal position for the deposition setup. By depositing 10k shots of Ni-free STO on all four sites (I-IV) and repeating the process with Ni-doped STO, followed by Ni-free STO, a radially uniform thin film thickness is achieved.

(a)

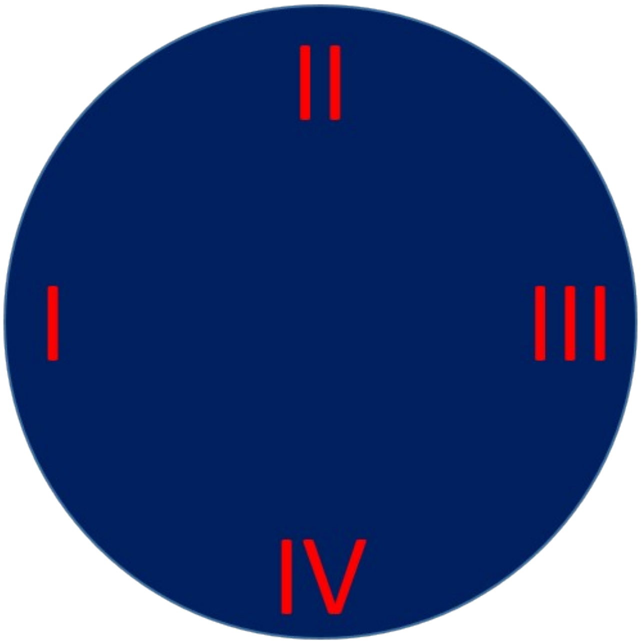

Figure S16: The different deposition positions in the PLD setup utilized in this work. The roman numbering marks the approximate position of the target underneath the wafer, leading to a radially uniform thin film thickness.

Supplementary Note 2:

TEM samples were prepared using two different techniques. The first, and utilized for most samples, is the grinding technique. The grinding occurred on an Allied MultiPrep^{TM} polishing system, where a wedge was formed. The thinned samples were mounted on TEM sample holders made of copper. Subsequently, the samples were polished with the PIPS II system (Precision Ion Polishing System II, Gatan Inc.). The disadvantage of this method is the possibility of re-deposition of part of the samples.

Another technique used was the Focused Ion Beam technique, where no mechanical grinding is utilized. Here, ions are used to create a TEM sample and thin it.

Supplementary Note 3:

A FEI Titan G2 60-300 instrument was used to perform STEM and high-resolution EELS. A CEOS DCOR corrector for the probe forming lenses, a Wien-filter monochromator, a Gatan 965 Quantum EELS spectrometer and a FEI Super-X EDS detector were installed on the instrument. The instrument was operated at 300 kV acceleration voltage for structural and chemical characterization, with a probe convergence of 21 mrad. The HAADF, DF4, and DF2 have 101, 22, and 9 mrad, respectively, as inner collection semi-angle.
